# The Proposed National Patho-Biological Laboratory

**Published:** 1888-12

**Authors:** 


BUFFALO MEDICAL AND SURGICAL JOURNAL
A MONTHLY REVIEW OF MEDICINE AND SURGERY.

EDITORS:
THOS. LOTHROP, M. D., - - - W. W. POTTER, M. D.

All communications, whether of a literary or business character, should be addressed to the editors: 284 Franklin Street, Buffalo, N. Y.

### Editorial.

### THE PROPOSED NATIONAL PATHO-BIOLOGICAL LABORATORY.

The proposition to establish a national patho-biological laboratory will excite a keen interest not only in medical and scientific circles, but in the large class whose financial success is, more or less, affected by the existence of contagious disease. We have had abundant occasion to see during the past few months how extensive sections of our Southern belt can suffer severely from this cause. Production is stopped, commerce hampered, the general well-being of communities lessened, and their natural healthful growth seriously checked, as the result of even a limited visitation of the yellow-fever scourge. It is impossible to calculate the loss to the State of Florida from the diversion of travel and immigration alone, which is the inevitable sequel of her recent misfortunes.

Still more extended geographically are the vast interests of our stock raisers, so frequently imperiled or crippled by the prevalence of infectious complaints among the more important varieties of domestic animals. The question of contagion is, in fact, one which touches closely many leading phases of our life as a people, such as foreign and internal travel and commerce, the management of our municipalities, the development of entire sections of territory, and the prosperity of our enormous agricultural interests, dependent invariably on the propagation and preservation of animal life.

It is, therefore, eminently fitting that the subject should be taken up earnestly and exhaustively by our national government. Excellent work, on a limited scale, has been done by individuals, State institutions, and some government departments, notably in the Agricultural Department and by the National Board of Health, before the limits of the latter's usefulness was so restricted by legislative interference.

The question is, however, too far-reaching territorially to be successfully managed by State action, and too closely related to the general welfare for satisfactory and thorough treatment in any existing Washington department.

The bill as proposed meets the wants of the case in many respects. Ample provision is made for the study of all problems from both the biological and chemical sides, and the sharp separation of the two divisions for the study of human and animal diseases would undoubtedly stimulate healthful rivalry, while not preventing helpful co-operation. The proposition to place the institution under the control of the Supervising Surgeon-General of the Marine Hospital Service may possibly be subject to criticism, chiefly in view of the zoo-pathological features of the work. It would, however, be difficult to select any one official in the public service thoroughly versed in all the proposed phases of investigation, and it is probable that the Surgeon-General M. H. S. is better placed for exercising a general supervision over the laboratory than any of his scientific colleagues.

We should not underestimate the value of the proposed institution, in rendering our political Capital more and more a center of advanced scientific research and higher learning. European nations find manifold advantages in multiplying the number of such establishments at their capitals, centralizing and consolidating the work of specialists, and attracting the talented youth of distant lands. Washington has already made a beginning in this direction, but it is yet far behind such cities as London, Paris, Berlin, Vienna, St. Petersburg, and even the residences of many of the minor German States.

The sum required to execute the provisions of the bill is certainly modest. It is but a drop in the bucket, compared with the vast draft on human effort and material resources in our land, due to the existence of contagious diseases—diseases which, as experience gives us every reason to believe, may ultimately be brought completely within man's control.

As a powerful factor in bringing about this result, we do not hesitate to recognize in the projected institute an object worthy of the cordial and earnest support of the medical profession; and it is our hope that sufficient intelligent and proper influence may be brought to bear upon our national legislators to ensure an early passage of the bill.

We publish in this issue of the JOURNAL a copy of the bill that is proposed on this subject, that our readers may judge of its merits for themselves.

